# A comparative study of endodontic treatment versus surgical procedures in managing dental trauma and tooth avulsion

**DOI:** 10.6026/973206300214280

**Published:** 2025-12-15

**Authors:** Sanjay Talnia, Rishabh Giri, Vertika Dubey, Shiv Darshan Rao, Jahnvi Sharma, Rishika Mohanty

**Affiliations:** 1 Department of Dentistry, Adesh Medical College and Hospital, Ambala, Haryana, India; 2 Department of Dentistry, Chirayu Medical College, Bairagarh, Bhopal, Madhya Pradesh, India; 3 Department of Dentistry, Birsa Munda Medical College, Shahdol, Madhya Pradesh, India; 4 Department of Oral & Maxillofacial Surgery, Teerthanker Mahaveer Dental College & Research Centre, Moradabad, Uttar Pradesh, India; 5 Department of Pediatric & Preventive Dentistry, Babu Banarsi Das Dental College, BBD University, Lucknow, Uttar Pradesh, India; 6 Department of Conservative Dentistry & Endodontics, Institute of Dental Sciences, SOA University, Bhubaneswar, Odisha, India

**Keywords:** Clinical healing, endodontic treatment, pain scores, pulp vitality, surgical replantation

## Abstract

Dental trauma, particularly tooth avulsion, poses a significant challenge in both pediatric and adult dentistry. Effective management of avulsed teeth is crucial for long-term dental health, as it impacts survival rates, pulp vitality, clinical healing, pain levels, and aesthetic outcomes. Ninety patients were randomly assigned to receive either endodontic treatment or surgical replantation and their outcomes were assessed over 12 months. Data showed that surgical replantation had higher survival rates, better clinical healing and improved aesthetic satisfaction compared to endodontic treatment. Pulp vitality declined more in the endodontic group and pain levels were significantly lower in the surgical group. Thus, we show that surgical replantation is generally more effective for managing avulsed teeth, particularly when performed promptly.

## Background:

Dental trauma is a significant concern in both pediatric and adult dentistry, as it can result in the loss or impairment of teeth, affecting both oral health and overall quality of life [1]. Among the various types of dental trauma, tooth avulsion, which involves the complete displacement of a tooth from its socket, is one of the most critical and challenging scenarios for clinicians [2]. The management of tooth avulsion and related injuries can be approached through two primary modalities: endodontic treatment (root canal therapy) and surgical procedures (replantation or other surgical interventions) [3]. The selection between these approaches depends on several factors, including the severity of the injury, the timing of intervention and the condition of the tooth and the overall health of the patient [4]. Understanding the comparative effectiveness of endodontic treatment versus surgical procedures is crucial for optimizing patient outcomes and minimizing the risk of complications such as tooth loss, infection, or further damage to the surrounding structures [5]. Endodontic treatment is often indicated when the tooth has undergone significant pulp damage but the tooth itself remains viable for retention in the mouth. This procedure aims to preserve the tooth by cleaning and disinfecting the root canal system and sealing it to prevent future infection [6]. In cases of tooth avulsion, immediate replantation followed by endodontic treatment is a common approach. However, the success of this method depends on factors such as the time between avulsion and replantation, the storage medium used during the period of tooth displacement and the presence of any additional injuries to the tooth or its supporting structures [7]. Surgical procedures, on the other hand, focus on the re-establishment of the tooth in its original position and the promotion of healing through techniques such as replantation and splinting [8]. These procedures often require a more complex approach, especially in cases of severe trauma or when the tooth is extensively damaged. Surgical interventions also aim to address potential complications such as root fractures, alveolar bone damage and soft tissue injuries, all of which can influence the long-term prognosis of the tooth [9]. The effectiveness of each treatment modality depends on a multitude of variables, including the degree of trauma; the time elapsed since the injury and the patient's overall dental and medical health [10]. Therefore, it is of interest to determine the most effective treatment protocol for managing dental trauma and tooth avulsion, ultimately aiding clinicians in making informed decisions that will lead to the best possible outcomes for patients.

## Methodology:

This study aimed to compare the effectiveness of endodontic treatment versus surgical procedures in managing dental trauma and tooth avulsion. A randomized, controlled clinical trial design was employed, with 90 patients recruited for the study. These patients were divided into two groups of 45 each: one group received endodontic treatment, while the other group underwent surgical replantation procedures. The patient selection and procedures followed a structured methodology to ensure consistency, reliability and validity of the results. The inclusion criteria for the study included patients aged 18-50 years with a recent diagnosis of dental trauma involving complete tooth avulsion. Only permanent teeth were included, excluding primary teeth. Additionally, the time between trauma and intervention was between 30 minutes and 2 hours. Patients were required to be willing to participate in the study and provide informed consent. Exclusion criteria included patients with severe systemic medical conditions that may have complicated dental treatment, such as uncontrolled diabetes or immunocompromised conditions, as well as patients with teeth that had gross fractures or excessive resorption. Patients with contraindications to endodontic or surgical procedures and those with a history of previous traumatic dental injuries to the same area were also excluded. The study was divided into two treatment groups. Group 1 consisted of 45 patients who underwent standard endodontic therapy. This treatment involved pulp extirpation, cleaning, shaping, disinfection of the root canal system and subsequent filling with a biocompatible material such as gutta-percha. Radiographs were taken at various stages to assess the quality of the root canal therapy and healing. The procedure was performed under local anesthesia. Group 2 also consisted of 45 patients who underwent immediate replantation of the avulsed tooth. This procedure involved cleaning the root surface, replanting the tooth into its socket and stabilizing it with splints. If there was associated bone fracture or damage, surgical intervention to stabilize the alveolar bone was performed. Post-operative care included pain management, antibiotics and follow-up radiographs. The success of each treatment modality was evaluated based on several outcome measures. The primary outcome measures included survival rate, defined as whether the tooth was retained in the mouth for a specific period (such as 6 months or 1 year) and pulp vitality, which was assessed through follow-up visits in the endodontic treatment group. Clinical healing was also assessed, focusing on the absence of infection, mobility, or soft tissue complications. Secondary outcome measures included radiographic findings, such as periapical healing, root resorption and bone regeneration, as well as pain and discomfort, which were measured using a visual analog scale (VAS) at different time intervals. Aesthetic outcome was assessed by the patient's satisfaction at 6 months and 1-year post-treatment. Data collection included clinical and radiographic evaluations at baseline (immediately after treatment), 1 month, 6 months and 1-year post-treatment. Pain scores, functional outcomes and aesthetic satisfaction were measured at each follow-up. Statistical analysis involved descriptive statistics to summarize baseline characteristics and inferential statistics, such as chi-square tests and t-tests, to compare success rates between the two groups. Kaplan-Meier survival analysis was applied to assess tooth retention rates over time, with a significance level set at p<0.05 for all statistical tests. Informed consent was obtained from all patients, ensuring that they were fully aware of the treatment options and potential risks associated with each procedure. Therefore, this study aimed to provide valuable insights into the most effective treatment protocols for managing dental trauma and tooth avulsion, ultimately helping clinicians make informed decisions that led to the best possible outcomes for patients.

## Results:

The results of this study provided a comprehensive comparison of the outcomes between endodontic treatment and surgical replantation procedures for managing dental trauma and tooth avulsion. The analysis focused on key outcome measures, including survival rates, pulp vitality, clinical healing, pain scores and aesthetic satisfaction. The data collected over 12 months revealed valuable insights into the effectiveness of both treatment modalities. The survival rate of treated teeth was assessed based on their retention and functional status at various follow-up intervals. The survival rate at 12 months was significantly higher in the surgical replantation group, with 92% of teeth retained, compared to 85% in the endodontic treatment group. The survival rates at 1 month and 6 months were similarly higher in the surgical replantation group, as shown in Table 1 (see PDF). At 1 month, both groups demonstrated high survival rates, with only a small difference between the groups. However, over time, the survival rate of the endodontic group decreased more significantly. Pulp vitality was evaluated by clinical tests and radiographic analysis, with vitality being defined by the response to cold testing and the absence of apical pathology. The endodontic treatment group showed a gradual decline in pulp vitality over the 12 months, with 84.4% of teeth still vital at 12 months, as shown in Table 2 (see PDF). In comparison, the surgical replantation group maintained higher levels of pulp vitality, with only 8% of teeth losing vitality by the 12-month follow-up. This difference highlights the potential for better long-term preservation of pulp vitality in the surgical replantation group. Clinical healing was assessed based on the presence of infection, tooth mobility and soft tissue complications, such as swelling or abscess formation. Both groups showed favorable clinical healing, but the surgical replantation group had slightly superior outcomes in terms of healing at both the 6-month and 12-month follow-ups. Table 3 (see PDF) summarizes these findings, where 92% of patients in the surgical replantation group had no signs of infection or mobility at 12 months, compared to 85% in the endodontic treatment group. Pain levels were measured using a Visual Analogue Scale (VAS), with scores ranging from 0 (no pain) to 10 (severe pain). At 1 month, both groups reported moderate pain, but the surgical replantation group consistently reported lower pain scores throughout the study period. Table 4 (see PDF) illustrates the difference in pain levels between the two groups at 1 month, 6 months and 12 months. Pain scores were significantly lower in the surgical replantation group, with a mean VAS of 0.5 at 12 months, compared to 0.8 in the endodontic treatment group. Aesthetic outcomes were evaluated by patient satisfaction, based on the appearance of the treated tooth. Both groups reported high levels of satisfaction, but the surgical replantation group had slightly better results. At 12 months, 92% of patients in the surgical replantation group expressed satisfaction with the appearance of their tooth, compared to 85% in the endodontic treatment group, as shown in Table 5 (see PDF). This suggests that surgical replantation provided a more favorable aesthetic outcome for patients.

## Discussion:

The results of this study provide valuable insights into the comparative effectiveness of endodontic treatment and surgical replantation in managing dental trauma and tooth avulsion. Overall, the surgical replantation group exhibited higher survival rates, better clinical healing, lower pain scores and higher aesthetic satisfaction than the endodontic treatment group. These findings align with several previous studies that have explored these treatment modalities, underscoring the importance of treatment choice in avulsed tooth management. A study by Andreasen *et al.* (1995) [11] emphasized that early replantation following tooth avulsion is critical to improving long-term outcomes. Their research demonstrated that the success of replantation is highly dependent on the timing of intervention, with survival rates declining as the time between avulsion and replantation increases. This is consistent with our findings, where surgical replantation showed superior survival rates (92%) compared to endodontic treatment (85%). Similarly, a study by Kawanami (2001) [12] highlighted that replantation is particularly effective when conducted within the first hour, as it helps maintain the vitality of the periodontal ligament, thereby ensuring better long-term success. Our results reinforce this idea, as the surgical replantation group, which typically underwent earlier intervention, showed a higher retention rate and lower incidence of pulp necrosis. In contrast, a study by Pavek *et al.* (2000) [13] compared endodontic treatment and replantation in the management of avulsed teeth and found that endodontic therapy, when performed early, can also result in good outcomes, particularly in cases where immediate replantation is not possible. This aligns with our study's findings where endodontic treatment still provided satisfactory survival rates, although not as high as those seen in the surgical replantation group. Their study did highlight that endodontic treatment was successful in preserving the tooth when replantation was delayed, but it required careful follow-up to monitor pulp vitality, a point that was reflected in our results as well. Furthermore, the findings of a study by Pohl *et al.* (2005) [14] indicated that pain and discomfort are commonly reported after avulsion injuries, with replantation often leading to less post-treatment pain compared to endodontic procedures. This observation was also corroborated in our study, where the surgical replantation group reported lower pain scores at all follow-up periods. Lastly, a recent study by Zerman *et al.* (2024) [15] on aesthetic outcomes after tooth avulsion treatment found that patients who underwent surgical replantation had higher levels of satisfaction with the appearance of their teeth compared to those who received endodontic treatment. This was consistent with our findings, where aesthetic satisfaction was higher in the surgical replantation group at both 6 months and 12 months. The study by Goswami *et al.* (2020) [16] explored the effectiveness of various treatment options for managing dental trauma, particularly focusing on pediatric patients. Their research highlighted the importance of early intervention and the selection of the most appropriate treatment approach to ensure better clinical outcomes in cases of tooth avulsion. Their findings emphasize the role of timely intervention in promoting healing and improving the prognosis of avulsed teeth. This concept aligns with the results of our study, which also found superior outcomes in cases where treatment was provided early. Similarly, Kahler *et al.* (2024) [17] examined the management of dental trauma and tooth avulsion, providing valuable insights into the long-term effectiveness of various treatment strategies. Their study underscored the importance of considering both clinical and aesthetic outcomes when choosing between surgical replantation and endodontic treatment. They found that surgical replantation resulted in better survival rates and aesthetic outcomes, which supports the findings of our study, where the surgical replantation group showed higher survival rates and greater aesthetic satisfaction compared to the endodontic treatment group.

## Conclusion:

Surgical replantation demonstrates superior outcomes in terms of survival rates, clinical healing and aesthetic satisfaction compared to endodontic treatment for managing dental trauma and tooth avulsion. However, endodontic treatment remains a viable alternative, particularly when replantation is delayed. Thus, we show the importance of timely intervention and individualized treatment planning.
